# Dr. William D. McGill

**Published:** 1911-12

**Authors:** 


Dr. William D. McGill, a graduate of Harvard Homeopathic, Cleveland, 1880, and a resident of Buffalo, died at Eden, N. Y., November 25, 1911.

